# Applying Relatedness to Explain Learning Outcomes of STEM Maker Activities

**DOI:** 10.3389/fpsyg.2021.800569

**Published:** 2022-01-17

**Authors:** Xiaojing Weng, Thomas K. F. Chiu, Morris S. Y. Jong

**Affiliations:** ^1^Department of Curriculum and Instruction, Faculty of Education, The Chinese University of Hong Kong, Hong Kong, Hong Kong SAR, China; ^2^Centre for Learning Sciences and Technologies, Faculty of Education, The Chinese University of Hong Kong, Hong Kong, Hong Kong SAR, China

**Keywords:** STEM education, maker, relatedness, mentoring, real-world problem, 21st century skills

## Abstract

A growing interest has been observed among K-12 school educators to incorporate maker pedagogy into science, technology, engineering, and mathematics (STEM) education to engage students in the design and making process. Both cognitive engagement and emotional engagement of students can be promoted through satisfying the psychological need of relatedness that concerns a sense of connection and belonging. How to support relatedness would influence the effective development of students’ cognitive competencies, namely creativity and critical thinking, and non-cognitive characteristics, namely interest and identity. Therefore, the present study investigated how two relatedness support strategies—real-world problems (RWP) and mentoring influence the development of student’s STEM-related cognitive competencies and non-cognitive characteristics in STEM marker activities. We implemented a 7-week intervention study with three classes of Grade 9 students (aged 13–15 years) in Hong Kong (*n* = 95). Three intervention conditions were designed in the experiment, comprising textbook problem (TBP), RWP, and RWP with mentoring (RWPM). Our analysis showed that (i) the differences in creativity among the three groups were non-significant, (ii) the RWP and RWPM groups showed stronger critical thinking than the TBP group, and (iii) the RWPM group exhibited stronger STEM interest and identity than the other two groups. This study revealed the effectiveness of adopting RWP strategy in developing secondary students’ perceived cognitive competencies (e.g., creativity and critical thinking) and the feasibility of employing a mentoring mechanism for cultivating learners’ perceived non-cognitive characteristics (e.g., STEM identity and interest). Hence, we also offered practical suggestions for teachers.

## Introduction

After the release of the Make magazine in 2005 and the hosting of the first Maker Faire in 2006, the maker movement started to gain momentum into becoming a worldwide phenomenon (Sang and Simpson, 2019). This movement is a cultural trend focused on creating makers rather than consumers of products in the 21st century (Marshall and Harron, 2018) and advocates for creativity, excitement, and innovation (Bevan et al., 2015; Papadakis, 2021). Within this movement, individuals can use different tools and materials to present their ideas through physical products such as prototypes and artifacts that they feel are relevant and interesting. This maker-centered approach has been applied in science, technology, engineering, and mathematics (STEM) learning and teaching (Honey et al., 2014; Godhe et al., 2019) primarily by means of after-school or extracurricular activities, and in libraries, museums, or playgrounds.

The incorporation of maker education in STEM learning and teaching is considered as contemporary pedagogy that embraces collaboration, experimentation, and open-endedness (Nemorin and Selwyn, 2017; Godhe et al., 2019); however, this pedagogy is less established in classrooms and relatively new to most schools and teachers (Honey et al., 2014). How to use maker approach to better engage in STEM learning warrants further exploration. Student engagement can be motivated by supporting a psychological need—relatedness (a sense of connection and belonging)—posited by Self-determination theory (SDT) that is a motivation theory (Ryan and Deci, 2020). To satisfy the need for relatedness, teachers could use authentic or real-world examples and assignments to establish students’ perceived relevance and connection of the learning materials (Hung, 2016), and adopt mentorship programs to foster stronger students’ belonging by developing student–expert relationships (Simões and Alarcão, 2014). Accordingly, real-world problems (RWPs) and mentoring would better motivate student engagement than textbook problem (TBP) in STEM maker activities.

Incorporating RWPs into STEM learning activities is more likely to expose students to authentic problems, which connects content with their daily life and lead to greater cognitive and emotional engagement. The inclusion of authentic problems will require students to solve ill-defined problems that are complex and cognitively challenging (English and Mousoulides, 2015; English, 2016; Woods and Hsu, 2020). The solving process requires students to process volumes of different information and brainstorm solutions. Students are expected to collaborate creatively, perform critical thinking, and communicate to propose, devise, and evaluate solutions to address the problems. The relevance of and connection with RWPs encourage students to be aware of the choice of solutions they arrive at and how their choices fit into a societal context in which they feel loved and care for, i.e., support the need for relatedness (Hung, 2016). Therefore, how maker pedagogy support relatedness could determine how effectively students’ cognitive competencies such as creativity and critical thinking can be developed. Moreover, mentorship programs support the need for relatedness, allow students to avail themselves of student–expert relationships, and to connect with their mentors (Simões and Alarcão, 2014). As mentees, students engage in different interactions with mentors in course of their learning process (Tofel-Grehl et al., 2017). They receive mentor’s guidance to shape their ideas, endorsement of their choices, and recognition of their efforts and works. This process could facilitate positive non-cognitive characteristics, such as interest and identity development in a social environment (Schlegel et al., 2019; Nganga et al., 2020). Relationships with mentors could influence the positive development of students’ STEM interest and identity (Nganga et al., 2020). However, the adoption of mentoring that supports relatedness (Simões and Alarcão, 2014) for promoting these non-cognitive characteristics has been less discussed in K-12 education (Honey et al., 2014), because mentorship programs require numerous resources involving an enormous number of school students.

In sum, further research to understand how the pedagogical designs of maker education to use RWPs and mentoring to support relatedness (Simões and Alarcão, 2014; Hung, 2016) in K-12 formal schooling settings is required (Honey et al., 2014; Godhe et al., 2019). Creativity, critical thinking, interest, and identity are the four major outcomes of STEM education in K-12 STEM education (Honey et al., 2014; Johnson et al., 2020). These vital features are necessary for the identification of young children as STEM makers and for their adaptation to the future society (Chiu et al., 2020; Chiu and Lim, 2020). Accordingly, the present study aimed to explain how RWPs and mentoring influence the development of student’s cognitive competencies and non-cognitive characteristics from a different perspective: relatedness.

First, we present our conceptual framework, and critically discussed previous studies on cognitive competencies and non-cognitive characteristics as learning outcomes of maker pedagogy. Then, we state the goal of this study, and its three research questions, following by describing research design and procedure. Final, we present the results answering the questions, and a discussion of the findings with limitations.

## Literature Review

### Maker Pedagogy in STEM Education

Making is an essential human activity because “we must make, create, and express ourselves to feel whole” (Hatch, 2014, p. 11). The concept of making is well-accepted as minds-on and hands-on learning in STEM education. Many schools have adopted making as a pedagogical approach with strong emphasis on designing, doing, and creating to nurture students to be creative STEM thinkers (Halverson and Sheridan, 2014; Godhe et al., 2019; Suh et al., 2020).

Creativity refers to the ability to generate multiple solutions to a problem through divergent thinking rather than one solution through convergent thinking (Daly et al., 2014). Maker pedagogy may allow students to explore a problem as a team, exchange ideas among their members, and build and rebuild their ideas. Furthermore, creativity acts as a vital tool that helps students put their new ideas into practice and develop their creative competence through innovation (Papadakis, 2021; Xia et al., 2021). Literature suggests that STEM maker projects can effectively cultivate students’ creativity in the school context (Saorín et al., 2017; Searle et al., 2018). For example, students can work in groups and share their ideas with peers to make creatively designed dolls using 3D printing (Saorín et al., 2017). Similarly, high school students in a circuit creation workshop had new ideas for their artifacts of electronic textiles when they were given opportunities to brainstorm different solutions and receive ideas from others (Searle et al., 2018). In general, maker pedagogy fosters a creative culture and play a positive role in helping students imagine innovative new possibilities.

Maker pedagogy, including hands-on activities and inquiry-based learning, is fundamentally linked to experiential learning (Honey et al., 2014; Suh et al., 2020). This type of pedagogy is based on the constructivist paradigm that students learn through experiencing and reflecting on those experiences (Martin, 2015). Teachers set the stage for students with real-life challenges, and students identify problems, design different possible solutions, and propose evaluation methods related to the challenges (Gettings, 2016; Chiu et al., 2021b). The students engage in in-depth investigations with materials, objects, and ideas and draw meaning and understanding from their experiences in a fun manner, in order to solve challenges with originality and imagination. The entire solving process includes observations and reflections. They are required to recruit and coordinate personal, social, and material resources for meaningful learning participation, i.e., they should have abilities to engage with sophisticated practices (Brahms and Crowley, 2016). Therefore, making as learning process for young children requires their access and evolving relationship to teacher/mentor assistance and expertise. These suggest that relatedness satisfaction would become more crucial in solving RWPs in STEM making activities.

### Textbook Problem, Real-World Problem, Mentoring, and Relatedness in Self-Determination Theory

Maker approach is less established pedagogy being adopted in STEM classrooms (Honey et al., 2014). How student engagement in maker learning activities affect the development of cognitive competencies such as collaboration, creativity, and critical thinking and non-cognitive characteristics such as enthusiasm, interest, and identity remains unclear (Honey et al., 2014; Martin, 2015). Student engagement can be motivated by satisfying relatedness, a psychological need that refers to a sense of connection and belonging suggested in Self-determination theory (SDT) (Ryan and Deci, 2020). In the course of learning, students’ behavioral, cognitive and emotional engagement can be speculated by the relatedness of the learning context (Reeve, 2013; Chiu, 2021b,c; Chiu et al., 2021a). It is because desirable teacher-student relationships can promote students’ active participation in maker activities (i.e., behavioral engagement), stimulate their positive feelings when pursuing the activities (i.e., emotional engagement), and make them more confident in accomplishing the challenging tasks therein (i.e., cognitive engagement) (Chiu, 2021b,c). In this paper, TBPs refer to the problem are not in real-life context. Accordingly, compared to TBPs, RWPs and mentoring are approaches to support need for relatedness: Students would perceive stronger relevance and connection of the learning materials when using RWPs, and would develop stronger belongings and student–expert relationships when adopting mentoring approach in learning activities (English and Mousoulides, 2015; Chiu, 2021a; Chiu et al., 2021a).

In TBPs students are often given specific ill-defined problems (Hanif et al., 2019). However, students may not be required to identify issues and formulate their own problems in real-life context. They may follow teachers’ instruction, and replicate peers’ ideas to solve the TBPs that are less complicated and authentic than RWPs. Therefore, students would find TBPs and their solutions less relevant, connected, and ownerships (Lee et al., 2013). In RWPs, students are afforded opportunities to make connections between STEM concepts and real-life applications (Achmetli et al., 2019), such as building own surgical masks and designing own smart home appliances. Their lived experiences and outcomes from authentic context-based activities and tasks provide some encouragement in working through these issues (Lee et al., 2013). Allowing students to identify their own issue in an RWPs and suggest solutions develop a strong sense of belonging and ownership to the activities and tasks (Lee et al., 2013; Achmetli et al., 2019), which support the need for relatedness. Accordingly, TBPs and RWPs are both problem-based learning, and enhance students’ creativity (Hanif et al., 2019).

As discussed in last section, young children require their access and evolving relationship to teacher/mentor assistance and expertise for making activities, particularly for more complex problems, i.e., RWPS. Effective mentorship occurs when mentors and mentees develop trust, and identify with and authentically engage with one another (Stoeger et al., 2013; Tenenbaum et al., 2014). They can speak freely and express ideas without concerns for interpersonal comfort (Chiu, 2021b; Chiu et al., 2021a). The effective mentor-mentee relationships allow students to interact and be connected to their methods, which lead to an enhanced sense of relatedness and effectiveness in the mentee (Ryan and Deci, 2020). Mentees benefit from engaging with mentors who share expert knowledge and experiences.

Accordingly, RWPs and mentoring that support relatedness, develop better 21st century cognitive competencies such as creativity, and critical thinking, and more positive non-cognitive learning outcomes such as interest, and identity (Ryan and Deci, 2020; Chiu, 2021b).

### Critical Thinking and Real-World Problems

Critical thinking is the ability to think clearly and rationally to form a judgment. It requires students to analyze complex problems, make connections across different disciplines, and evaluate solutions (Hu et al., 2020). STEM disciplines often work together seamlessly in the real world. Introducing students to RWPs enables them to see the connection between “what’s inside” and “what’s outside” their classroom; they see that their learning is more than giving correct answer to tests and getting good grades. The RWPs build bridges beyond the classroom by connecting discipline content with daily life, offering learning opportunities with local and global communities, showing everyday applications of learning, and engaging students in authentic learning that are meaningful. These result in greater cognitive engagement that facilitates their critical thinking advancements (Hu et al., 2020). For instance, Hollman et al. (2019) reported that 645 secondary school students engaged with real world problems that improved their critical thinking; English and Mousoulides (2015) real world problems facilitated the development of Grade 6 students’ critical thinking. It may be because real world problems are cognitively challenging (English and Mousoulides, 2015; Woods and Hsu, 2020) and require students to carefully consider all the decisions concerning their solutions and how the solutions fit into societal contexts (English, 2016). To solve these problems, students need to collaborate creatively and think critically to propose possible solutions. These RWPs are more relevant to students than textbook problems, for example, building a bridge to connect a highway. The relevance of RWPs can support students need for relatedness by connecting problems to their life and own understanding. i.e., it encourages students to perform comprehensive research, seek advice and feedback from others, and employ their critical thinking. In addition to cognitive competencies (e.g., critical thinking), maker pedagogy should aim to cultivate students’ non-cognitive characteristics such as STEM interest and identity (Fasso and Knight, 2020).

### STEM Interest and Identity and Mentoring

STEM interest and identity are two critical predictors of students’ higher studies and career plans in STEM-related fields (Hurk et al., 2019). This is because they reflect individuals’ self-images and enables them to derive personal meaning from their endeavors in the STEM field. Hence, fostering students’ STEM interest and identity are two major learning outcomes of maker pedagogy in STEM classrooms (Cannady et al., 2014; Honey et al., 2014; Hurk et al., 2019). Interest is often characterized in terms of curiosity, persistence, and resourcefulness (Hidi and Renninger, 2006), and develops over time—it germinates when people’s attention is triggered and develops through voluntary engagement or re-engagement (Renninger and Hidi, 2011). The development of students interest can be guided (Renninger, 2010). Interested students have stronger feelings of self-efficacy and can cultivate better self-regulated behaviors to persevere on with challenging tasks (Hidi and Ainley, 2008). Maker pedagogy that promotes student autonomy in terms of allowing them to decide which solutions to arrive at is postulated to increase student motivation for STEM learning because of the sense of personal ownership that students feel throughout the design, making, and testing process. Furthermore, maker pedagogy has been demonstrated to be effective in increasing students’ interest and engagement in science and math classes (Gerber et al., 2012; Honey et al., 2014; Holmlund et al., 2018).

Identity refers to who one is and how one is recognized by others. A person’s identity is shaped by how they are recognized in a given context, with particular interests, talents, and ways of being in particular social contexts (Honey et al., 2014; Goos and Bennison, 2019). Identity development is a matter of finding oneself by matching one’s talents and potential with available social roles (Goos and Bennison, 2019). Recognition from other people, which determines the credibility and value of a person’s performance, is a necessary component of identity formation. A few studies have suggested that integrated experience from project- and problem-based learning can promote identity development. For example, Hachey et al. (2021) suggested that the flexibility and openness of STEM learning activities that allow students to define their own designs are influential factors in fostering a STEM-related identity. However, this was a very preliminary finding (Honey et al., 2014). Maker pedagogy alone may not effectively foster STEM identity.

Introducing role models or mentors in STEM education can enhance students’ STEM interest and identity development (Honey et al., 2014; Nelson et al., 2017; Huvard et al., 2020). In mentorship programs, students often engage in three types of interactions with mentors: shareability, tangibility, and aesthetic (Tofel-Grehl et al., 2017). Shareability enables students to obtain access to various resources, better understand problems, and make creative prototypes under their mentors’ guidance (Kreider et al., 2018). Tangibility allows students to forge personal connections to internalize their interest and identity (Fasso and Knight, 2020). Aesthetic allows for students’ efforts and/or works to be endorsed by mentors, and this acceptance further helps students to recognize their identity in a social environment. These interactions trigger and sustain interest and lead to the development of a stronger identity (Tofel-Grehl et al., 2017). Studies have suggested that the mentoring approach can better develop students’ STEM identity. For example, Ladeji-Osias et al. (2018) and Musavi et al. (2018) revealed under-represented minority students developed a stronger STEM identity and a stronger desire to pursue STEM careers in a mentorship program; Pinkard et al. (2017) found that middle school female student-mentees developed a stronger interest and identity toward STEM. In summary, adding mentoring to maker pedagogy would lead to the development of stronger STEM interest and identity.

## The Present Study

Supporting the need for relatedness that better cognitively and emotionally engages students in learning may better develop critical thinking, and enable the positive formation of interest and identity. This paper constitutes an interventional study that investigates whether RWPs and mentorships in maker pedagogy can influence the development of learners’ cognitive competencies, namely creativity and critical thinking, and non-cognitive characteristics, namely interest and identity, in K-12 school classrooms. To achieve the research goal, testing the intervention is the main research task; therefore, we employed an experimental method with three interventional conditions, namely TBP, RWP, and RWP with mentoring (RWPM). The TBP group used an ill-defined problem in non-real-life context; the RWP group used an ill-defined problem in real-life context; the RWP group used an ill-defined problem in real-life context and mentors. Accordingly, the following research questions were proposed:

RQ1. Are there differences among the three groups in terms of perceived cognitive competencies?
RQ2. Are there differences among the three groups in terms of perceived non-cognitive characteristics?
RQ3. Do the three groups have stronger cognitive competencies and non-cognitive characteristics?

We hypothesized the following: no significant difference exists in terms of creativity among the three groups (H1); the RWP and RWPM groups develop stronger critical thinking skills (H2); the RWPM group develop stronger STEM interest (H3) and STEM identity (H4); and the three groups develop stronger cognitive competencies and non-cognitive characteristics (H5).

## Methodology

### Participants

The participants comprised 95 Secondary Three students, aged between 13 and 15 years, and three teachers from three different schools in Hong Kong with similar academic performance. The schools were randomly assigned to three interventional conditions: TBP (*n* = 32), RWP (*n* = 31), and RWPM (*n* = 32). The average teaching experience of the teachers was 5 years. Eight undergraduate student mentors pursuing STEM-related majors were recruited to facilitate the intervention.

### Procedures

We got the ethical approval from university, and first obtained the consent of the students and their parents. Before the intervention, we conducted two 3-h workshops on Arduino kits for making pedagogy in STEM education for all the teachers, conducted two co-planning sessions with the teachers, recruited all the student mentors, and administered the pre-questionnaire among the students. The intervention was conducted in 14 lessons for 7 weeks. During the intervention, all the students learned in group of three to four. Student in TBP used a textbook-like problem—assembling Arduino kits to make different traffic light systems for their learning. The RWP group learned through a real-life problem—traffic light system for a specific area, they identified their own areas or occasions and proposed solutions. Students in RWPM used the same real-life problem for their project as students in RWP with mentoring from eight mentors, i.e., each student team was guided by one mentor. The students finished the post-questionnaire in the last lesson. Table 1 outlines the pedagogies of the three groups. In Week 1, the teachers introduced Arduino I/O circuit board and computer programming interface to students. From Week 2 to Week 4, the teachers acted as facilitators to foster student understanding of STEM prerequisite knowledge by asking questions, giving feedback, and monitoring learning progress. The prerequisite knowledge of Science, Technology, Mathematics were electricity (Physics), coding and ratios respectively. Engineering design process was adopted. From Week 5–7, different student groups learned with different problems—TBP, RWP and RWPM. The problem in RWP and RWPM groups is a design task. In RWPM, all the students worked collaboratively, guided and facilitated by their mentors, to design solutions for their problems. The mentors encouraged their mentees, helped with problem solving, and used active-listening techniques, and served as a guide for mentees’ behavior, values, and attitudes. The teachers joined the discussions if necessary. Compared with the teachers, the mentors had much more intimate and frequent communications with students.

**TABLE 1:** Intervention.

Time and theme	Activity examples
Week 1	• Teachers introduced Arduino I/O circuit board and computer programming interface to students.
Meet Arduino and my first Arduino program	• Student groups ° Learned to connect the Arduino board to the laptop with the provided USB cable. ° Worked in groups to follow instructions on a worksheet to finish their first Arduino program (lighting up the LED). ° Explored other extension programs.

Week 2	• Teachers acted as facilitators.
Basic knowledge 1	• Student groups ° Modified the program and observed the changes in the brightness of the LED lights. ° Created extension programs to control the LEDs at three different brightness levels by using the push buttons.

Week 3	• Teachers acted as facilitators.
Basic knowledge 2	• Student groups ° Explored a way to connect the variable resistor. ° Adjusted the variable resistor to observe the number changes. ° Designed a variable by recording the input voltage. • Teachers guided students to review the mathematical knowledge about proportions. • Student groups ° Designed a new variable to control the brightness of the LEDs and use variable resistance to change it. ° Tried to write extension programs according to the instructions on the worksheet.

Week 4	• Teachers acted as facilitators.
Basic knowledge 3	• Student groups ° Wrote/rewrote a new program to control the brightness of LEDs according to the brightness of the surrounding environment. ° Tried to write extension programs according to the instructions on the worksheet.

Week 5–7	• Teachers acted as facilitators.
Problem-solving with Arduino	• In the TBP groups, teachers introduced and reintroduced different suggested traffic light systems as problems in Weeks 1 and 5.
	• In the RWP and TWPM groups, teachers explained an existing situation of public traffic light systems in the community. For example, the public traffic light is turned on by workers at 6 pm every evening with a fixed brightness level. Students introduced and reintroduced a real-world problem—propose solutions for specific areas or occasions in Weeks 1 and 5.
	• Students in TWPM groups were introduced to their mentors in Week 2.

### Measures

Questionnaires were used to measure four variables in the before and after the intervention, namely creativity, critical thinking, STEM interest and identity. Each of the variables was measured by three items using a 5-point Likert scale that solicited ordinal responses from 1 (strongly disagree) to 5 (strongly agree). All the questions were adapted from the previously published instruments.

Creativity and critical thinking were measured using the items from the study of Kelley et al. (2019) with good reliabilities (e.g., creativity α = 0.75, critical thinking α = 0.88). The participants of Kelly and colleagues were K-12 students, which fit the participant of this study. The items were modified to fit the research goals designed for creativity, for example, I am confident in my ability to understand how knowledge or insights might transfer to other situations or contexts, and I am confident in my ability to elaborate and improve on ideas. The examples of the items that evaluated critical thinking were: I am confident in my ability to evaluate reasoning and evidence that support an argument, and I am confident in my ability to justify choices of evaluation criteria.

STEM interest was measured using the items of STEM Semantics Survey Scales designed by Tyler-Wood et al. (2010) with acceptable reliability (e.g., α of Science, Math, Engineering, Technology, and STEM as a career interest ranged from 0.84 to 0.93). Their assessment target was participants’ perceptions of scientific disciplines, which was similar to this study purpose. The items were revised to match our research context; for instance, I find STEM fascinating, I find STEM exciting, and I find STEM appealing.

STEM identity was evaluated using the three items of identification adopted in the study of Godwin (2016): the constructs of competence, self-recognition, and recognition by others. Godwin’s study investigated students’ identity in STEM-related domain. The original items were adapted to fit our research situations through statements such as I am able to do well in activities that involve STEM, I see myself as a STEM individual, and My best friends see me as a STEM individual.

Moreover, our analysis demonstrated that the reliability of all variables ranged from 0.78 to 0.95 in this study and was acceptable [Cronbach alpha (CA) ≥ 0.7, see Table 2; Taber, 2018].

**TABLE 2:** Scale characteristics.

Scales	CA in pre-questionnaire	CA in post-questionnaire
Creativity	0.83	0.78
Critical thinking	0.88	0.95
STEM interest	0.93	0.88
STEM identity	0.90	0.92

### Analysis Approaches

A one-way ANCOVA was utilized to examine how the three pedagogical designs affect participants’ perceived cognitive competencies and non-cognitive characteristics while controlling the pre-questionnaire scores, to answer RQ1 and RQ2. The pedagogical design was the independent variable and the pre- and post-questionnaire scores were covariates and dependent variables, respectively. To answer RQ3, paired-sample *t* tests were conducted to examine whether perceived cognitive competencies and non-cognitive characteristics improved in the three groups by comparing the mean scores of their pre- and post- questionnaires.

## Results

### Descriptive Statistics

Table 3 presents the descriptive statistics of creativity, critical thinking, STEM interest, and identity in both the two questionnaires. Our analyses revealed there was no significant difference among the pre-questionnaire scores of creativity, *F* (2, 94) = 0.10, *p* = 0.91; critical thinking, *F* (2, 94) = 0.57, *p* = 0.57; STEM interest, *F* (2, 94) = 0.38, *p* = 0.69; and STEM identity, *F* (2, 94) = 1.81, *p* = 0.17. These scores showed that the three experimental groups perceived cognitive competencies and non-cognitive characteristics in similar way before the intervention.

**TABLE 3:** Descriptive statistics and paired *t*-test for pre- and post-questionnaires.

		Pre-questionnaire		Post-questionnaire		Paired *t*-test
Groups	Variables	Mean	SD	Mean	SD	
TBP (*N* = 32)	Creativity	2.79	0.59	4.30	0.43	−12.15***
	Critical thinking	2.90	0.84	3.22	0.74	0.12
	STEM interest	2.85	0.80	3.84	0.56	−7.11***
	STEM identity	2.89	0.83	3.19	0.69	−3.26***
RWP (*N* = 31)	Creativity	2.85	0.72	4.27	0.49	−9.37***
	Critical thinking	3.08	0.65	3.77	0.82	−3.62***
	STEM interest	2.87	0.85	3.85	0.65	−6.95***
	STEM identity	2.67	0.56	3.18	0.38	−5.36***
RWPM (*N* = 32)	Creativity	2.78	0.64	4.22	0.57	−8.90***
	Critical thinking	2.98	0.46	4.10	1.03	−5.59***
	STEM interest	3.02	0.88	4.60	0.68	−8.50***
	STEM identity	3.02	0.80	4.60	0.45	−13.12***

*p < 0.05; **p < 0.01; and ***p < 0.001.

### RQ1: Differences in Perceived Cognitive Competencies

#### Creativity

According to our Levene’s test, homogeneity existed with *F* = 1.17, *p* = 0.31. One-way ANCOVA showed no significant differences in students’ creativity among the three groups in the post-questionnaire by excluding the effect of their pre-questionnaire scores, *F* (2, 91) = 0.22 and *p* = 0.80 (see Table 4).

**TABLE 4:** ANCOVA results of post-questionnaire for the three groups.

Scales	Groups	*N*	Adjusted mean	SE	*F*	*Post hoc*
	TBP	32	4.30	0.09	0.22	
Creativity	RWP	31	4.27	0.09		
	RWPM	32	4.22	0.09		
	TBP	32	3.21	0.16	8.41***	(1) < (2)
Critical thinking	RWP	31	3.78	0.16		
	RWPM	32	4.10	0.16		(1) < (3)
	TBP	32	3.86	0.11	14.86***	(1) < (3)
STEM interest	RWP	31	3.86	0.11		(2) < (3)
	RWPM	32	4.58	0.11		
	TBP	32	3.18	0.07	104.12***	(1) < (3)
STEM identity	RWP	31	3.27	0.08		(2) < (3)
	RWPM	32	4.53	0.07		

*p < 0.05, **p < 0.01, and ***p < 0.001.

#### Critical Thinking

Levene’s test was conducted and the assumption was validated, *F* = 2.93, *p* = 0.06. The analysis revealed significant differences in students’ critical thinking after the intervention, with *F* (2, 91) = 8.41, *p* < 0.001. A *post hoc* comparison showed no significant improvements for the RWP group (adjusted mean = 3.78) over the TBP group (adjusted mean = 3.21, p = 0.04) and the RWPM group (adjusted mean = 4.10) over the TBP group (*p* < 0.001).

### RQ2: Differences in Perceived Non-cognitive Characteristics

#### STEM Interest

Homogeneity existed with *F* = 1.29, *p* = 0.28. The analysis was significant, with *F* (2, 91) = 14.86, *p* < 0.001. A *post hoc* comparison indicated that the RWPM group (adjusted mean = 4.58) significantly outperformed the TBP group in the post-questionnaire (adjusted mean = 3.86, *p* < 0.001), and the RWP group (adjusted mean = 3.86, *p* < 0.001).

#### STEM Identity

Homogeneity was proved by Levene’s test, *F* = 2.68, *p* = 0.07. The analysis was significant with *F* (2, 91) = 104.12, *p* < 0.001. Significant differences were observed in the three groups’ STEM identity after the intervention. A *post hoc* comparison revealed that the post-questionnaire scores of the RWPM group (adjusted mean = 4.53) were significantly higher than that of the TBP group (adjusted mean = 3.18, *p* < 0.001), and the RWP group (adjusted mean = 3.27, *p* < 0.001).

### RQ3: Improvement of Perceived Cognitive Competencies and Non-cognitive Characteristics

In response to RQ3, this study further examined the perceived improvement of the three groups before and after the intervention by conducting paired-sample *t* tests, see Table 3. All the participants showed significant improvement in creativity, with TBP group (*t* = −12.15, *p* < 0.001), RWP group (*t* = −9.37, *p* < 0.001), and RWPM group (*t* = −8.90, *p* < 0.001). Furthermore, the RWP and RWPM groups achieved significant improvement in critical thinking, with *t* = −3.62, *p* < 0.001 and *t* = −5.59, *p* < 0.001, respectively. However, the TBP group showed no significant improvement in this dimension with *t* = −1.60, *p* = 0.12.

STEM interest and identity of the three groups were significantly different before and after the intervention. Students of the TBP group (*t* = −7.11, *p* < 0.001), RWP group (*t* = −6.95, *p* < 0.001) and RWPM groups (*t* = −8.50, *p* < 0.001) showed perceived stronger STEM interest. Similarly, the TBP group (*t* = −3.26, *p* < 0.001), RWP group (*t* = −5.36, *p* < 0.001) and RWPM group (*t* = −13.12, *p* < 0.001) reported significantly stronger STEM identity.

## Discussion

The intervention conducted in this paper was designed to examine how different pedagogical designs supporting the need for relatedness, i.e., real-life problems and mentoring, influence the development of cognitive competencies and non-cognitive characteristics. This study presents four major findings; accordingly, three practical suggestions are presented for practitioners and instructional designers.

First, as predicted, maker pedagogy exerted an impact on nurturing students’ creative competence. In line with the studies of Saorín et al. (2017) and Searle et al. (2018), our results highlighted maker activities improved creativity for all three groups (H1 and H5). These results imply that (i) maker activities provided students with learning opportunities that facilitated their creative competence as they implemented their ideas through creating artifacts and (ii) the relevance of problems pertaining to maker pedagogy and the relationships with and guidance from a more experienced or knowledgeable person did not better foster the competence of students. Furthermore, students were found to better develop their creative skills through innovation by putting their new ideas into practice that encouraged divergent thinking when students are encouraged to envision and suggest multiple solutions (Xia et al., 2021).

Second, the critical thinking of all the three groups showed improvement and the students who solved RWPs outperformed those who solved TBPs (H2 and H5). This is in alignment with earlier studies that advocated the use of RWPs in design and maker activities (English and Mousoulides, 2015; Hollman et al., 2019). A plausible explanation is that students felt connected and relevant, and were motivated to explore and brainstorm more solutions for the problems (Ryan and Deci, 2020). The processes of coming up solutions encouraged students to evaluate their ideas for developing better critical thinking skills (Hu et al., 2020). The connection also encourages students to carefully and thoughtfully consider and decide their choices fit into a societal context in which they feel loved and care for. Another explanation is about, when addressing RWPs, students process a large amount of information that is contradictory and potentially unreliable and have to make connections across different disciplines to arrive at solutions. In this learning process, they are needed to carefully read and critically process information to consolidate their ideas (English and Mousoulides, 2015). Therefore, the relevance and complexity of the problems influence how students can think clearly, rationally, and critically to form a decision.

Third, students in all three groups developed more positive interest and identity toward STEM, and students in the mentorship group particularly developed much stronger interest and identity than the other two groups that did not involve mentors (H3, H4, and H5). These results are supported by studies that suggest that engaging role models or mentors in STEM learning can better cultivate students’ STEM interest and identity (Honey et al., 2014; Pinkard et al., 2017; Ladeji-Osias et al., 2018; Musavi et al., 2018; Huvard et al., 2020). These findings also imply that positive interest and identity toward STEM are developed in a social context (Renninger and Hidi, 2011; Goos and Bennison, 2019). Both interest and identity reflect how students view themselves (self-images and endeavors) and how they feel others perceive them (recognitions from others) in the context of STEM. Therefore, their development requires the internalization process of need satisfaction for relatedness (Ryan and Deci, 2020). This internalization process is catalyzed by engaging mentors or role models in maker learning due to the different interactions with mentors, namely shareability, tangibility, and aesthetic (Tofel-Grehl et al., 2017). In this study, the mentors used active-listening techniques to establish and maintain positive mentor-mentee relationships by encouraging their mentees and helping with problem solving. The students can speak freely and express ideas, resulting in interpersonal comfort and more intimate and frequent communications. Accordingly, offering positive student–expert relationships in maker pedagogy can more effectively foster students’ STEM interest and identity.

The final finding suggests that in maker pedagogy, supporting relatedness by using student–expert relationships and RWPS types can determine the effectiveness of the development of learners’ non-cognitive characteristics, namely STEM interest and identity (third finding), and cognitive competency, that is critical thinking. Therefore, non-cognitive characteristics in STEM are influenced by interaction with role models but not by analyzing problems and building artifacts. Cognitive competencies that involve extensive thinking require deeper learning, and these attributes can be affected only through the processing, application, and emulation of knowledge.

## Practical Suggestions

Creativity, critical thinking, interest, and identity are the significant outcomes of STEM education (Honey et al., 2014). Our study shows how STEM learning can be designed using relatedness support to foster the four outcomes in a formal curriculum. Our first suggestion is to encourage STEM curriculum leaders should emphasize the development of student STEM interest and identity by offering a promising pedagogical approach—satisfying the need for relatedness. We suggest that teachers should use learning strategies that make students feel connected and loved, such as students’ favorable raw materials for making, community- and school-based problems and involvement of more knowledgeable persons (e.g., role models or mentors). Our second suggestion is that teachers should consider student-mentor relationships when planning and designing their STEM maker lessons or projects. To achieve that, teacher professional programs on how to co-teach with mentors should be offered because this approach is new to most STEM teachers. Finally, we recommend that teachers should use scaffolding ideas to help students achieve the four learning outcomes (Chiu et al., 2021b). Because mentorship programs require numerous resources, schools may not be able to sustainably provide such programs. Teachers can use TBPs to build students’ basic making skills, followed by RWPs and mentorship programs.

## Conclusion and Limitations

Overall, this study suggested supporting relatedness can influence students’ cognitive competencies and non-cognitive characteristics in STEM learning. This study had limitations and five of them are noted here. First, while this study appears to support how to satisfy the need for relatedness affect the effectiveness of learning outcomes (Nganga et al., 2020), additional studies validating these findings are required. The results of the present intervention could also be extended by additional studies by including other problem types (local and global), other experts such as STEM professionals, and other cognitive and non-cognitive skills. Second, this study did not consider how long interest and identity can persist. Further research should be conducted using longitudinal design to examine the long-term effects of different maker pedagogies. Third, how students collaborate with peers or mentors in making may have impact on the outcomes of this study. Future studies are suggested to examine how different collaborative learning affect the outcomes of the students. The fourth limitation are the definition of creativity. This study adopted the definition from an engineering education (Daly et al., 2014); however, creativity can be defined in terms of three key aspects—fluency, flexibility, and originality. Future studies should adopt these aspects in measuring student creativity with comprehensive approach. The final limitation of the study is that the invention was conducted over different sessions. Environmental factors may influence student motivation for learning, leading to differences between conditions. The intervention should be conducted in parallel sessions by future studies.

## Data Availability Statement

The raw data supporting the conclusions of this article will be made available by the authors, without undue reservation.

## Ethics Statement

The studies involving human participants were reviewed and approved by Faculty of Education, The Chinese University of Hong Kong. Written informed consent to participate in this study was provided by the participants’ legal guardian/next of kin.

## Author Contributions

XW analyzed the data, drafted and revised the manuscript. TC conceptualized this study, managed the research project, and revised the work. MJ revised and proofread the article. All authors approved the submission.

## Conflict of Interest

The authors declare that the research was conducted in the absence of any commercial or financial relationships that could be construed as a potential conflict of interest.

## Publisher’s Note

All claims expressed in this article are solely those of the authors and do not necessarily represent those of their affiliated organizations, or those of the publisher, the editors and the reviewers. Any product that may be evaluated in this article, or claim that may be made by its manufacturer, is not guaranteed or endorsed by the publisher.
